# Prediction of Radiation Pneumonitis With Dose Distribution: A Convolutional Neural Network (CNN) Based Model

**DOI:** 10.3389/fonc.2019.01500

**Published:** 2020-01-31

**Authors:** Bin Liang, Yuan Tian, Xinyuan Chen, Hui Yan, Lingling Yan, Tao Zhang, Zongmei Zhou, Lvhua Wang, Jianrong Dai

**Affiliations:** Department of Radiation Oncology, National Cancer Center/National Clinical Research Center for Cancer/Cancer Hospital, Chinese Academy of Medical Sciences and Peking Union Medical College, Beijing, China

**Keywords:** convolutional neural network, dose distribution, pneumonitis prediction, dosiomics, deep learning

## Abstract

Radiation pneumonitis (RP) is one of the major side effects of thoracic radiotherapy. The aim of this study is to build a dose distribution based prediction model, and investigate the correlation of RP incidence and high-order features of dose distribution. A convolution 3D (C3D) neural network was used to construct the prediction model. The C3D network was pre-trained for action recognition. The dose distribution was used as input of the prediction model. With the C3D network, the convolution operation was performed in 3D space. The guided gradient-weighted class activation map (grad-CAM) was utilized to locate the regions of dose distribution which were strongly correlated with grade≥2 and grade<2 RP cases, respectively. The features learned by the convolution filters were generated with gradient ascend to understand the deep network. The performance of the C3D prediction model was evaluated by comparing with three multivariate logistic regression (LR) prediction models, which used the dosimetric, normal tissue complication probability (NTCP) or dosiomics factors as input, respectively. All the prediction models were validated using 70 non-small cell lung cancer (NSCLC) patients treated with volumetric modulated arc therapy (VMAT). The area under curve (AUC) of C3D prediction model was 0.842. While the AUC of the three LR models were 0.676, 0.744 and 0.782, respectively. The guided grad-CAM indicated that the low-dose region of contralateral lung and high-dose region of ipsilateral lung were strongly correlated with the grade≥2 and grade<2 RP cases, respectively. The features learned by shallow filters were simple and globally consistent, and of monotonous color. The features of deeper filters displayed more complicated pattern, which was hard or impossible to give strict mathematical definition. In conclusion, we built a C3D model for thoracic radiotherapy toxicity prediction. The results demonstrate its performance is superior over the classical LR models. In addition, CNN also offers a new perspective to further understand RP incidence.

## Introduction

Radiation pneumonitis (RP) is one of the most common side effects of thoracic radiotherapy. Accurate prediction model is desired to safely irradiate the tumor target without increasing the risk of RP. The simple and straightforward dosimetric factors, such as the mean lung dose (MLD) and dose volume factors (the volume receiving dose greater than xGy, V_x_), have been proven to be closely related with RP incidence, but the conclusions drawn from published studies differ from each other (1–4). Compared with dosimetric factors, the normal tissue complication probability (NTCP) factors have shown better prediction capability (5–7) and smaller disagreement between different institutions (8). The improvement can be possibly explained by the utilization of more information of the dose distribution. For instance, V_x_ can be interpreted as a discrete point on the dose volume histogram (DVH) curve. On the other hand, the NTCP factor utilizes all information of the DVH curve. But still, the spatial information of dose distribution is not utilized.

For medical images, such as CT, MRI, and PET, the spatial distribution of intensity levels is visually perceived as image “textures.” The recently emerged radiomics method extracts the texture features from medical images, and studies their correlation with therapeutic responses (9–14). The dosiomics method was developed by applying the framework of radiomics on dose distribution, i.e., to extract texture features from dose distribution (15–17). Previous, we utilized the dosiomics method for RP incidence prediction (18). The results demonstrate that the dosiomics features outperform both the dosimetric and NTCP factors.

Essentially, the radiomics (dosiomics) method first extracts features from medical images (dose distribution) and then adopts classical machine learning (ML) approaches, such as logistic regression (LR) and random forests, to construct the prediction model. Although a large number of features are extracted, high-order “subtle” features may possibly be neglected in this process. With the increasing computational power of modern computer, the recently developed deep learning (DL) method (19) directly uses the raw data as input, and utilizes conventional neural network (CNN) to reveal the subtle feature hidden in original raw data. The CNN-based DL method overwhelmingly outperforms classical ML methods, and has been applied to pattern recognition, medical image analysis, and bioinformatics etc. Zhen et al. first applied CNN on the unfolded 2D dose distribution of rectum surface for toxicity prediction (17). Ibragimov et al. extended to 3D dose distribution for hepatobiliary toxicity prediction (20). Both studies validated the feasibility to use CNN-based DL method for toxicity prediction after radiotherapy.

In this study, we applied the convolution 3D (C3D) network (21) for RP incidence prediction. The C3D network was pre-trained with UCF101 video dataset (22) for the task of action recognition. The 3D dose distribution within total lungs was used as the input. The performance of C3D models was compared with three dosimetric, NTCP or dosiomics factors based models. The guided grad-CAM method (23) was used to illustrate the discriminative regions. In addition, we also investigated the correlation of RP incidence and high-order features of dose distribution.

## Methods and Materials

In this section, we first briefly introduced the patient dataset, then presented the network architecture, feature illustration and training strategy in details, and finally described the comparison against LR models based on the dosimetric, NTCP, or dosiomics factors.

### Patient Data

We used the same patient dataset as previous study (18). The dataset includes 70 non-small cell lung cancer (NSCLC) patients treated with 6MV volumetric modulated arc therapy (VMAT) in our institution from 2013 to 2016. The radiotherapy treatment plans were designed via Pinnacle treatment planning system (v 9.0). The dose was prescribed to 95% of the planning target (PTV) volume. The slice spacing of planning CT and grid spacing of dose calculation was 5 mm and 4 mm^3^, respectively. RP grade (ranging from 0 to 5) was evaluated according to Common Terminology Criteria for Adverse Events (CTCAE v3.0). The endpoint of all prediction models presented in this study is grade≥2 RP. For the sake of simplicity, the grade≥2 RP cases are referred as RP cases, and the grade 0 and 1 cases as none-RP cases in the following text.

### C3D Network

C3D network was designed for the task of video classification. The input of C3D network is 3D data volume (frame-volume) derived by stacking up video frames. The convolution and pooling operations are also performed in 3D. The tasks of video classification and RP prediction appear quite different from each other. But essentially, both tasks attempt to search for the discriminative features using 3D convolutions. Thus, the C3D network can be used for dose distribution based RP prediction.

Another consideration is that the C3D network comes with a set of well pre-trained parameters. The C3D network was pre-trained with UCF101 video dataset (22). UCF101 dataset contains 13320 video clips from 101 categories, which is sufficient to “feed” the complicated deep network. In (24), Zhang et al. proved that the fully connected (FC) layers of CNN are task-oriented, which act like a “firewall” and guarantee the generality of the features learned by convolution layers. Therefore, it is possible to transfer the pre-trained model when the source domain (frame-volume) is completely different from the target domain (dose distribution).

#### Architecture

**Figure 1:**
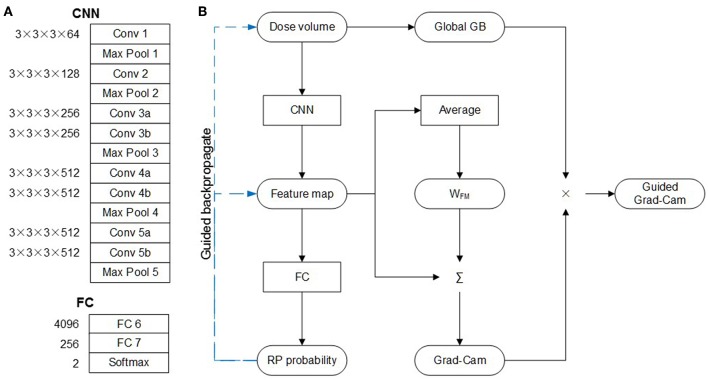
C3D network. **(A)** Architecture. **(B)** Guided Grad-Cam.

The architecture of C3D network is shown in Figure 1A, which is composed of five convolution layers followed by two fully connection (FC) layers. The kernel and weight dimension of the convolution and FC layers are also denoted in Figure 1A. The original network was designed to recognize 101 different actions in the video. For this study, the task is to predict the probability of the RP and none-RP cases, i.e., to discriminate the two cases. Thus, the dimensions of FC 6, FC 7, and softmax layers are compressed to 4,096 × 256, 256 × 256, and 256 × 2 for the sake of redundancy reduction.

#### Guided Grad-CAM and Convolution Filters

The guided gradient weighted class activation mapping (grad-CAM) method was utilized to illustrate the critical regions of dose distribution. As shown in Figure 1B, with dose volume as input, forward-propagation yields RP probability. If the network is interpreted as a highly complicated non-linear function, then back-propagation (GB) operation is the calculation of its gradient. The global GB is the gradient of RP probability to dose volume. And the guided GB neutralizes the negative rectified linear unit (ReLu) neuron to further suppress the noise. According to the definition of gradient, the guided GB locates the critical region of dose distribution, and the increase of dose in the critical region may lead to significant increase or decrease of RP probability. This means that the critical region is not discriminative for the RP and none-RP cases.

The discriminative region can be shown by the CAM method, which replaces the two FC layers with global average pooling (GAP) layer. The weight of GAP layer (W_FM_) is retrained for each class, which represents for the importance of corresponding feature map to particular class. Therefore, the weighted summation of W_FM_ and feature map highlights the discriminate region for the RP and none-RP cases, respectively. The grad-CAM method proves the mean gradient of FC layers is identical with W_FM_, thus saves the need of retraining. And the guided grad-CAM is calculated as Hadamard production of global GB and grad-CAM.

In addition, the features learned by each filter were also investigated. These features were represented by the input images which product the largest output when subjected to the convolution of corresponding filter. The input image with maximal output was derived by gradient ascent.

#### Training Strategy

Transfer learning was used to address the issue of dataset scarcity. The C3D network was pre-trained with the UCF101 dataset for the task of action recognition. The dose volume was first sampled to 64 × 64 × 64, and then copied to 64 × 64 × 64 × 3 to accommodate the architecture of C3D network. In order to prevent overfitting, the following strategies were adopted: 1. mini-batch size was set to 10; 2. only 1 epoch was used; 3. L2 regulation penalty were added on the weights of FC layers; and 4. a random dropout was added on FC 6 layer. For the issue of data imbalance, the RP cases in the training dataset were oversampled. And the training dataset was augmented by flipping along three directions. Three different strategies were tested: (1) training from scratch (Scratch), (2) training the FC layers only (FC), and (3) fine-tuning the convolution layers and training the FC layers simultaneously (Both). The learning rate of training and fine-tuning was set to 10^−3^ and 10^−4^, respectively.

### Multivariate LR Prediction Models

The C3D prediction model was evaluated by comparing with the three LR prediction models presented in our previous study (19). All the three models were built using multivariate LR, and the inputs were 1. dosimetric factors, 2. NTCP factors and 3. dosiomics factors, respectively. The dosimetric factors include V_5_, V_10_, V_15_, V_20_, and MLD. The NTCP factors are two sets of equivalent uniform dose (EUD) and NTCP factors of Lyman (25) and parallel/serial (PS) models (26). The dosiomics factors include 27 indices derived from the gray level co-occurrence matrix (GLCM) and 16 indices derived from gray level run length matrix (GLRLM). The calculation formulas can be referred in (27). All the features were calculated for the dose distribution within ipsilateral, contralateral and total lungs, separately. 1,000-time's bootstrap was used as training dataset, and the original dataset as validation dataset.

For all the three models, the number of predictors was fixed to 2 to avoid overfitting (28). All possible two-factor combinations were traversed. The combinations with mean Spearman correlation >0.8 or lower than −0.8 were excluded to prevent overfitting. And the optimal combination was determined as the combination with maximal mean training AUC. The final coefficient of LR model was determined as the median coefficient derived by training dataset.

Data pre-processing was implemented with Matlab software (MathWorks, Natick, MA). The C3D network was built and trained using tensorflow library in python language (v1.4.0) (29). The dosiomics features were extracted using the python pyradiomics package (v2.0.0) (27). Multivariate LR was implemented using the R stats package (v3.4.1) (30). Transparent reporting of a multivariable prediction model for individual prognosis or diagnosis (TRIPOD) statement can be found in the Supplementary Material.

## Results

Fifteen out of seventy patients (21.4%) were diagnosed with grade≥2 RP. The median prescription dose was 60Gy (ranging from 50Gy to 70Gy). Other clinical factors are listed in Table 1.

**Table 1:** Clinical factors.

**Characteristic**	**Value**
**Stage**	
I	4 (5.7%)
II	5 (7.1%)
III	53 (75.7%)
IV	8 (11.4%)
**Sex**	
Male	61 (87.1%)
Female	9 (12.9%)
**Age**	
Range	35-84
Mean ± Std.	61 ± 10
**Tumor location**	
Left	33 (47.1%)
Right	37 (52.9%)
**KPS**	
≤80	41 (58.6%)
>80	29 (41.4%)
**Concurrent chemotherapy**	
Yes	38 (54.3%)
No	32 (45.7%)
**Smoking history**	
Yes	60 (85.7%)
No	10 (14.3%)
**Prescription dose (Gy)**	
Single fraction	2.27 ± 0.85
Total	59.10 ± 5.67
**RP grade**	
≥2	15 (21.4%)
<2	55 (78.6%)

*KPS, Karnofsky performance status*.

### Prediction Ability Evaluation

**Figure 2:**
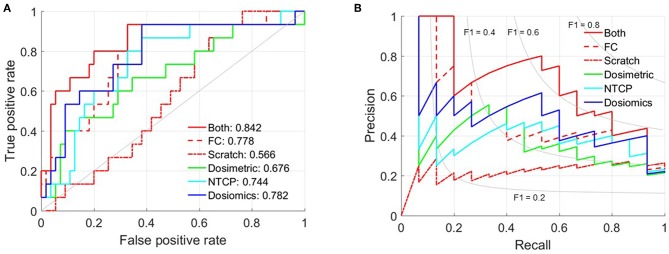
Prediction ability evaluation. **(A)** ROC curve. **(B)** Precision-Recall curve.

The mean AUC and standard deviation of 50-time random 10-fold cross validation is 0.566 ± 0.039 (Scratch), 0.778 ± 0.024 (FC) and 0.842 ± 0.049 (Both), respectively. And the 95% confidence interval is [0.533, 0.556], [0.772, 0.786], and [0.830, 0.863]. The *p*-value of paired t-test between Both against FC and Scratch is 1.238 × 10^−11^ and 1.031 × 10^−51^. Figure 2 shows the receiver operating characteristic (ROC) and precision-recall curves of the partition with mean AUC. The ROC and precision-recall curves of the three multivariate LR prediction models are also plotted in Figure 2. As the number of patient cases of this study is far from sufficient to feed the complicated C3D network, the prediction performance of training from scratch is only slightly better than random guess. The AUC of only training the FC layers indicates that directly using the set of parameters trained via the video dataset may not yield satisfactory result, since the 3D dose distribution is quite different from the frame-volume. Fine-tuning the convolution layers and training the FC layers simultaneously achieves the best prediction performance. The standard deviation of 50-time random 10-fold cross validation is relatively small, which validates the stability and also excludes the possibility of overfitting.

Table 2 lists the most significant predictors of the three multivariate LR prediction models. All the three prediction models extracts the two most significant predictors from different lung volumes. This is because the factors extracted from the same lung volume are strongly correlated, and the corresponding combinations are excluded to prevent overfitting. Detailed correlation analysis can be found in LeCun et al. (19). The C3D model outperforms the three LR models. The comparison of C3D model and the three LR models suggest that the prediction ability improves with the utilization of the information of the dose distribution.

**Table 2:** Multivariate LR prediction model^*^.

	**Significant factors**
Dosimetric	MLD^I^, ${\text{V}}_{5}^{\text{C}}$
NTCP	${\text{NTCP}}_{\text{PS}}^{\text{I}}$, ${\text{EUD}}_{\text{PS}}^{\text{C}}$
Dosiomics	GLCM^I^ contrast, GLRLM^T^ low gray level run emphasis

* *The superscripts “I,” “C” and “T” denote the features are extracted from ipsilateral, contralateral and total lungs, respectively*.

### Guided Grad-Cam and Convolution Filter

**Figure 3:**
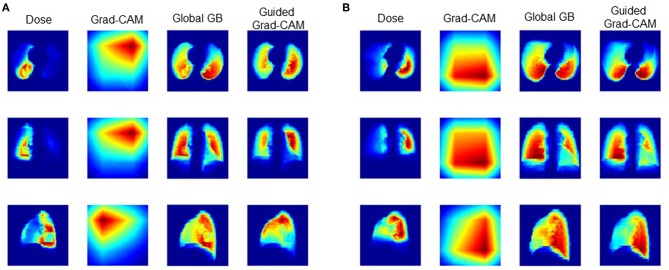
Comparison of RP and None-RP cases. **(A)** RP grade 2 case, **(B)** RP grade 0 case.

Figure 3 shows the dose distribution, grad-CAM, global GB and guided grad-CAM of the RP and none-RP cases, which were averaged along axial, sagittal, and coronal directions for the sake of clearance. The dose distribution within ipsilateral lung is higher than the dose distribution within contralateral lung. The global GB locates the regions that have greater influence on RP probability. As shown in Figure 3, both the high-dose region within ipsilateral lung and the low-dose region within contralateral lung are critical for RP prediction. The grad-CAM shows the discriminative region for the RP and none-RP cases. The low-dose region of contralateral lung and the high-dose region of ipsilateral lung are more correlated with the RP and none-RP cases, respectively.

**Figure 4:**
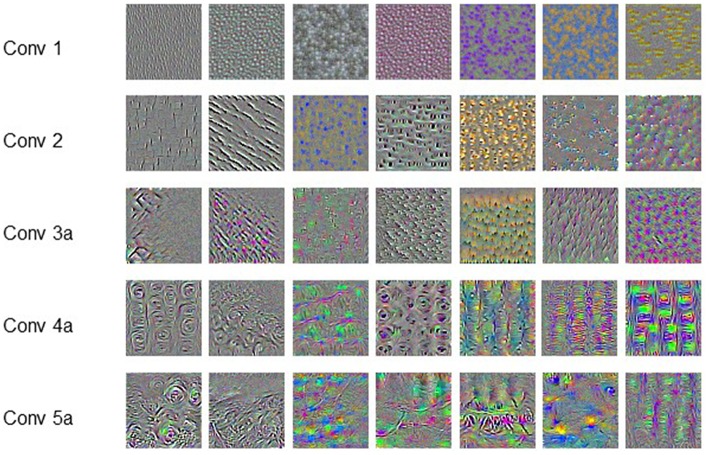
Convolution filters.

Figure 4 shows the representative features learned by the convolution filters, which are actually the input producing the largest output at corresponding filters. The pattern or texture of the features extracted by shallow filters is simple and globally consistent, and the color is also monotonous. As the filters getting deeper, the extracted features show more and more complicated pattern. The patterns are no longer globally consistent. Meanwhile, the features also become richer in color. It is hard or impossible to give strict mathematical definition of these high-order and unnatural features.

## Discussion

In our pervious study, we have validated that the predictive ability is positively correlated with the utilized information of the dose distribution. This conclusion is further validated by comparing the performance of dosiomics based prediction model and the C3D network presented in this study. Numerous features are extracted from the dose distribution by the dosiomics method, but do not include the high-order features learned by the C3D network as shown in Figure 4. The comparison also suggests that the CNN-based DL method outperforms the classical ML method on RP prediction, as it does in the field pattern recognition. The CNN-based DL method saves the need of hand-craft feature extraction and selection. The framework is more general than the dosiomics method, and is more suitable for radiotherapy outcomes, either positive (survival, control rate) or negative (normal tissue damage, complication).

Our results are consistent with published literatures on CNN-based DL based radiotherapy toxicity prediction. In Ibragimov et al. (20), reported that the AUC of CNN-based prediction model was 0.85. Zhen et al. reported that the AUC was improved to 0.89 (17). Zhen et al. also found that the VGG-16 network, which is pre-trained for image classification, can be “transferred” to 2D dose distribution based toxicity prediction. The best prediction performance is achieved by fine-tuning both the convolution and FC layers simultaneously, which agrees with the finding of this study.

The lack of large-scale labeled patient cases make it impossible to train the complicated CNN from scratch, and also increases the risk of overfitting. In Zhang et al. (24), proved that the FC layers are task-oriented, which act like a “firewall” and guarantee the generality of the features learned by convolution layers. Therefore, it is possible to transfer the pre-trained network when the source domain is completely different from the target domain.

The AUC is increased from 0.782 to 0.842 when switching from dosiomics method to DL technique. However, we see vague or no possibility of further improvements of any dose distribution based prediction models. This is because the dose distribution, even strongly correlated with radiotherapy toxicity incidence, is not the unique factor. Other clinical factors, such as the age and chemotherapy, are also associated with radiotherapy toxicity incidence.

Although the DL prediction model demonstrates satisfactory prediction capability, the understanding of the model is not clear. For now, we could only qualitatively assert that the spatial features of low-dose and high-dose regions are critical for RP cases and none-RP cases, respectively. The clinical meaning of the finding is unclear. Furthermore, how to utilize the finding for treatment plan design also needs to be further studied.

## Data Availability Statement

The raw data supporting the conclusions of this article will be made available by the authors, without undue reservation, to any qualified researcher.

## Ethics Statement

This study was carried out in accordance with the declaration of Helsinki and approved with exemption from informed consent by the independent ethics committee of cancer hospital, Chinese Academy of Medical Sciences (No. NCC2015 G-15).

## Author Contributions

BL, HY, and JD conceived the project and wrote the paper. XC, YT, and LY collected and analyzed the data. TZ, ZZ, and LW provided expert clinical knowledge. All authors edited the manuscript.

### Conflict of Interest

The authors declare that the research was conducted in the absence of any commercial or financial relationships that could be construed as a potential conflict of interest.
